# Social housing status impacts rhesus monkeys’ affective responding in classic threat processing tasks

**DOI:** 10.1038/s41598-022-08077-4

**Published:** 2022-03-09

**Authors:** Joey A. Charbonneau, David G. Amaral, Eliza Bliss-Moreau

**Affiliations:** 1 Neuroscience Graduate Program, University of California Davis, Davis, USA; 2 California National Primate Research Center, University of California Davis, Davis, USA; 3 The MIND Institute, University of California Davis School of Medicine, Davis, USA; 4 Department of Psychiatry and Behavioral Sciences, University of California Davis School of Medicine, Davis, USA; 5 Department of Psychology, University of California Davis, Davis, USA

**Keywords:** Emotion, Psychology, Animal behaviour

## Abstract

Individuals’ social contexts are broadly recognized to impact both their psychology and neurobiology. These effects are observed in people and in nonhuman animals who are the subjects for comparative and translational science. The social contexts in which monkeys are *reared* have long been recognized to have significant impacts on affective processing. Yet, the social contexts in which monkeys *live as adults* are often ignored and could have important consequences for interpreting findings, particularly those related to biopsychiatry and behavioral neuroscience studies. The extant nonhuman primate neuropsychological literature has historically tested individually-housed monkeys, creating a critical need to understand how social context might impact the outcomes of such experiments. We evaluated affective responding in adult rhesus monkeys living in four different social contexts using two classic threat processing tasks—a test of responsivity to objects and a test of responsivity to an unfamiliar human. These tasks have been commonly used in behavioral neuroscience for decades. Relative to monkeys with full access to a social partner, individually-housed monkeys had blunted reactivity to threat and monkeys who had limited contact with their partner were more reactive to some threatening stimuli. These results indicate that monkeys’ social housing contexts impact affective reactivity and point to the potential need to reconsider inferences drawn from prior studies in which the impacts of social context have not been considered.

## Introduction

Nonhuman animals have been used for decades in the service of understanding the biological mechanisms of phenomena like mood disorders and the basis of psychosocial behavior^1–4^. Nonhuman primates, specifically, are used extensively in psychological and psychiatric neuroscience research^5–7^ because of their psychological and biological homologies with humans^8–10^. Such studies often include neurobiological manipulations like lesioning particular brain regions or neural connections (e.g., see^11^ for a review of studies on the amygdala) and, more recently, genetic manipulations (e.g., see^12^ for a review). Typically, such experiments rely on a comparison between manipulated and unmanipulated animals (“control animals”), and the behavior or task performance of control animals is assumed to be “healthy” or species “normative”. Such approaches have historically ignored, or treated as inconsequential, aspects of the contexts in which animals are housed and tested. The human literature clearly demonstrates that isolated or limited social conditions impact affective processing, with social isolation and loneliness increasing levels of depression and introversion^13–17^. Following the logic that limiting social conditions could impact monkeys’ affective processing, it is possible that social contexts in which adults are living could influence how control animals behave and, thus, influence the interpretation of the effects of manipulations. Beyond this, the underlying source of social contextual effects may interact with experimental manipulations, complicating inferences drawn from ostensibly causal manipulations of biological mechanisms.

Neurobiological and psychobiological research conducted with animals has typically focused on psychosocial outcomes (e.g., anxiety-like behavior^18^) and measurements of specific behaviors thought to reflect these outcomes (e.g., freezing and cooing^5^). Such behavioral measures can be sensitive to the direct impact of, and interactions with, features of subjects’ environmental and social context (see^19^ for a review), sometimes in ways not immediately clear to experimenters. For example, in one rodent study the mere presence of a male vs. female experimenter (or even just the presence of clothing previously worn by a male experimenter) resulted in the differential inhibition of responses to painful stimuli^20^.

Social relationships and features of social context are widely recognized to play critical roles in human experience^21^ (see^22,23^ for reviews). In adult humans, social isolation and loneliness are associated with increased and earlier mortality^17,24^, chronic illness^25^, and depression^26^. Beyond physical health, social context and the strength of relationships with family and community correlate with evaluations of subjective wellbeing^27^. The interplay between human social relationships and psychosocial processes likely has deep evolutionary roots, seen in data gleaned with nonhuman primates, generally, and macaque monkeys, specifically. In macaques, rearing conditions interact significantly with genotypic features to produce behavioral differences^28^ and influence hormonal responses to threat^29^, among other differences (see^30^ for a review). Although much of the literature on the effects of social context in monkeys has focused on developmental context—that is, how infant and juvenile monkeys’ social environment influences psychosocial processing (see^31^ for an early review)—there is increasing evidence that variation in social housing impacts adult monkeys as well^32,33^.

In most cases, adult monkeys housed alone (without tactile contact with a social partner) are housed in rooms with other monkeys they can see and hear—so isolation in this case refers to physical features of social relationships. Often, experimenters will claim that these conditions do not actually reflect isolation, qualified by visual, auditory, and olfactory exposure to other monkeys (e.g.,^34,35^; see^36^ for a discussion). Despite this visual, auditory, and olfactory exposure, compared to socially-housed adult monkeys, individually-housed monkeys generate more aberrant behaviors, including self-injurious behaviors^37^, anxiety-like behaviors^18^, and depression-like behaviors^38^.

While the impact of adult social context on psychosocial outcomes is likely to be different from the impact of social context during rearing, the existing research on the impacts of adult social context is limited in scope. What studies exist are limited to assessing the impacts of social context on monkeys’ behavior at home (in their home cages)^18,37–44^ and physiological measures (e.g., cortisol levels, creatinine levels, absolute numbers of total T cells)^45–49^. While home cage behavior and physiology are critical measures for the successful maintenance of appropriate animal welfare in captive monkeys^50–52^, in order to best assess the potential impact of social context on past and future behavioral neuroscience experiments carried out on laboratory monkeys, it is essential that we directly assess the impact of this variable on the same outcome measures that these studies report, using task-based studies.

To assess the impact of varied social contexts on monkeys’ affective processing, we analyzed responses to threat in four cohorts of healthy adult rhesus macaques housed in various social configurations in the same laboratory, tested with identical experimental protocols, over several years. Monkeys completed a test of responsivity to ostensibly threatening objects (as in^53–56^) and a test of their responsivity to the presence of an unfamiliar human experimenter (the Human Intruder Test^57^). Importantly, the two tasks used here are widely used to characterize affective reactivity of monkeys’ natural or induced phenotypes (e.g., assessing the impact of amygdala^53,55,58,59^, hippocampus^60–62^, orbitofrontal cortex^63–65^, or anterior cingulate lesions^54^; assessing the impact of early adverse experience^66^; assessing differences in self-injurious monkeys^67^; determining neurobiological correlates of anxious temperament^68^) and are thought to provide a veridical representation of natural and induced variation in affective reactivity^54^. We capitalize upon methodological consistency across cohorts (including standardized protocols for observation, interrater reliability, etc.), but variation in housing condition as husbandry and laboratory standards changed across time. Subjects were individually-housed (no physical contact with any other monkey), grate-paired (access to a partner partially through a metal grate), intermittently-paired (access to a partner 7–8 h/day), or continuously-paired (access to a partner 24 h/day). Given the findings in the human mental health literature and nonhuman animal welfare literature, we expected social housing condition to impact animals’ affective behavior during tasks indexing threat sensitivity.

## Methods

Experimental procedures were approved by the University of California, Davis Institutional Animal Care and Use Committee, the ethics board overseeing nonhuman animal research at the university. Procedures were conducted in accordance with the National Institutes of Health guidelines for the use of animals in research. All procedures were carried out at the California National Primate Research Center (CNPRC). The present reporting follows the recommendations in the ARRIVE guidelines^69^.

### Subjects and living arrangements

Subjects were 22 adult male rhesus monkeys (*Macaca mulatta;* 5–10 years; Mean ± SD = 7.60 ± 1.37 years) that served as neurologically intact control animals for a series of behavioral neuroscience experiments over the course of 10 years (2001–2011) in the same laboratory^53,54,70–74^. They were born at CNPRC and raised by their mothers in large social groups in 0.2 hectare outdoor corrals, remaining there through adulthood. They were relocated to temperature-controlled indoor rooms (12-h light/dark cycle) where they were fed monkey chow (Ralston Purina, St. Louis, MO) twice daily supplemented with fresh fruit and vegetables twice per week and had ad libitum access to water and enrichment. Monkeys were housed in standard nonhuman primate caging (61 cm width × 66 cm depth × 81 cm height) in one of four possible configurations (see Table 1). Housing configurations were determined based on animal husbandry norms at the CNPRC at the time of testing and social compatibility with other study subjects or available partners. Some data were previously published (A1-6^53^; D1-7^54^) and the present report includes data from some monkeys involved in other previously published experiments (A1-6^70,73,74^; B1-4^72^; C1-5^70,71^). All monkeys in the present report were relocated to indoor housing between 15 and 29 months (see Table 1) prior to completing the tasks described here.

**Table 1:** Description of subjects.

Monkey	Housing configuration	ORT	ORT variant	ORT age	ORT time indoors	HIT	HIT variant	HIT age	HIT time indoors
A1	Individual	✓	1	8.98	1.50	✓	1	9.52	2.05
A2	Individual	✓	1	8.88	1.48	✓	1	9.42	2.02
A3	Individual	✓	1	9.03	1.43	✓	1	9.58	1.97
A4	Individual	✓	1	8.96	1.47	✓	1	9.50	2.01
A5	Individual	✓	1	7.02	1.42	✓	1	7.57	1.97
A6	Individual	✓	1	8.96	1.42	✓	1	9.50	1.96
B1	Grate	✓	2	9.91	1.29				
B2	Intermittent	✓	2	9.00	1.47				
B3	Intermittent	✓	2	7.03	1.47				
B4	Continuous	✓	2	8.92	2.47				
C1	Grate	✓	3	6.30	1.82	✓	2	6.52	2.04
C2	Grate	✓	3	8.16	1.78	✓	2	8.38	2.00
C3	Grate	✓	3	7.21	1.82	✓	2	7.42	2.04
C4	Grate	✓	3	6.22	1.82	✓	2	6.44	2.04
C5	Intermittent	✓	3	6.27	1.84	✓	2	6.48	2.06
D1	Intermittent	✓	4	6.16	1.86	✓	2	6.01	1.71
D2	Intermittent	✓	4	7.22	1.84	✓	2	7.07	1.68
D3	Intermittent	✓	4	7.36	1.84	✓	2	7.21	1.68
D4	Continuous (HIT), intermittent (ORT)	✓	4	6.24	1.95	✓	2	5.86	1.57
D5	Continuous	✓	4	7.23	1.70	✓	2	7.05	1.53
D6	Continuous	✓	4	7.16	1.72	✓	2	6.99	1.55
D7	Continuous	✓	4	6.37	1.95	✓	2	5.99	1.57

Housing configuration, variant used, age (in years), and time previously spent indoors (Time Indoors, in years) for ORT and HIT by monkey. The four caging configurations used were: Individual: only visual, auditory, and olfactory access to other monkeys; Grate: housed in adjacent individual cages adjoined by a grate which allowed visual and limited somatosensory access to a cagemate; Intermittent: housed in adjacent individual cages which permitted intermittent full access (5 or 7 days per week, 5–8 h per day) to a cagemate; Continuous: housed in adjacent individual cages which permitted continuous full access to a cagemate. Check marks in the ORT and HIT columns indicate that a given subject participated in ORT or HIT, respectively. Task structure varied slightly as described in the methods section with four different variants of ORT (1–4; see Table 2) and two variants of HIT (1,2).

Subjects were selected from large outdoor corrals at the CNPRC (total area: 0.2 hectare; 30.5 m width × 61 m depth × 2.44 m height) containing 60–120 monkeys following laboratory protocols to ensure that subjects were socially normal. This ensured that monkeys were raised in an environment most closely mirroring their natural environment as is possible in a captive setting. A series of 10 5-min focal observations were conducted to determine that all subjects were socially integrated and had no stereotypies or other abnormal behaviors prior to being enrolled in their respective experiments and relocated to indoor housing.

### Behavioral experiments

#### Object responsivity test

In the Object Responsivity Task (ORT), affective responses to threat were assessed by presenting monkeys with novel objects of varying complexity alongside a food reward. All 22 monkeys participated in one of four variations of the ORT (see Table 1). Only similar or identical trials were compared and only dependent variables that were identical across experiments were analyzed. The order of testing was counterbalanced across test day for each cohort and all testing occurred within the same time frame relative to meals (i.e., monkeys tested across variants should have been equally hungry). All monkeys were tested in the same lab care cage (80.01 cm × 83.32 cm × 101.6 cm) that was adapted to have a plexiglass front. The front had two vertical openings (25 cm × 5 cm), separated by 5.08 cm and centered 32.25 cm from the sides of the cage, which allowed the monkeys to interact with objects and retrieve the food reward. An opaque guillotine door lowered by a rope-and-pulley system blocked the plexiglass between trials. All trials were 30 s. Two observers sat diagonally to the right of the test cage (~ 2 m away) such that animals could view the observers when the opaque guillotine door was raised. A camera was also positioned directly in front of the test cage. No other monkeys were present in the room during testing.

Objects of varying complexity were used to evaluate changes in monkeys’ responses with variation in the affective value of stimuli (with simple objects being ostensibly less threatening than their complex counterparts; see discussion in^75^); complexity was varied across trials (see Table 2). All monkeys completed complex object trials and so the responses on these trials were compared. All monkeys were exposed to a combination of realistic (e.g., fake snake, alligator, lizard) and unrealistic (e.g., stuffed bear, stuffed lion, cheese grater) ostensibly threatening complex objects. Subject groups A, B, and D were exposed to both simple and complex object trials, so the responses on these trials were separately compared. All objects used were completely novel to the subjects.

**Table 2:** ORT trial structure by variant.

	Variant 1	Variant 2	Variant 3	Variant 4
Number of test days	6	12	5	10
Trial structure	(1) Food only (2) Food + simple (3) Food + medium (4) Food + complex (5) Food only	(1) Food only (2) Food + simple (3) Food + medium (4) Food + complex (5) Food only	(1) Food only (2) Food + complex (3) Food only (4) Food + complex (5) Food only (6) Food + complex	(1) Food only (2) Food + simple (3) Food only (4) Food + complex (5) Food only (6) Food + simple (7) Food only (8) Food + complex (9) Food only

Trial structure for each variant of ORT. Only trials comparable across variants were included in the analyses here. The stimuli used across variants were similar in size and structure and largely overlapping. In all four cases, complex stimuli were a combination of children’s toys (e.g., toy snake, lizard, bear) and household objects (e.g., lampshade, book, scrub brush). Simple stimuli were featureless objects of equivalent size and shape (e.g., blocks of wood or clay). An additional stimulus category (“medium”) was not included in the present analyses because they were only used in Variants 1 and 2. Stimuli in that category included modified forms of the complex stimuli which obscured eyes and other facial features or were simply the complex stimuli presented backwards so that monkeys could not see these features.

Behavioral data collection was carried out by trained observers using The Observer (Noldus, Sterling, VA, USA; versions 1 through 5, depending on sample) and included: (1) latency to retrieve the food reward; (2) frequency of food retrieval; (3) frequency of species-typical affective behaviors that were measured across all experiments (i.e., facial behaviors: lipsmack and grimace; see Table S1). As there is considerable variance in how individual animals deploy different affective behaviors and in the contextual meanings of these behaviors^76^, affective behaviors were summed within and across bins to generate a continuous index of affective reactivity for each animal during each trial (as in^77^). Additional behaviors were recorded across each test administration but were not present in all data sets. All observers were trained to the lab standard of inter-rater reliability greater than 85%. As the present analyses (comparing monkeys across social housing conditions) were not planned at the time of data collection or scoring for each experimental group, no experimenters involved were aware of group allocation on the basis of housing condition.

#### Human intruder test

In the Human Intruder Test (HIT), affective responses to threat were indexed by presenting monkeys with an unfamiliar human experimenter in various combinations of spatial orientation and distance from the subjects. Eighteen monkeys participated in one of two variants of the HIT (see Table 1; Monkeys B1-4 did not participate in the HIT). Monkeys were relocated to a testing room and isolated in a standard primate cage (60 cm × 75 cm × 75 cm). After a one minute acclimation period they were exposed to an unfamiliar male experimenter in four different conditions, as described previously by our group (e.g.^78^) and others (e.g.^79^). The four conditions were presented in the following order: (1) *profile far*: person facing 90° away from the cage at 1 m distance; (2) *profile near*: profile at 0.3 m; (3) *stare far*: direct stare at the animal at 1 m; (4) *stare near*: stare at 0.3 m. Trials were 30 s (Variant 1) and 60 s (Variant 2). Tests were repeated across 5 days.

Behavioral data were scored live using a 1/0 sampling method by trained observers (as in^53,54,78^). In Variant 1, six 5 s bins were used across 30 s trials. In Variant 2, six 10 s bins were used across 60 s trials. Behaviors that occurred within a bin received a score of 1 and behaviors that did not occur received a 0. To standardize across variants for data analysis, only the first three bins were used for monkeys in Variant 2 (totaling 30 s). Position in cage and six affect-related behaviors (i.e., lipsmack, grimace, threat, cage shake, tooth grind, and yawn) were scored (see Table S2). Position in cage was scored based upon the location of the monkey’s head in the cage (i.e., if the head was present in the front half of the cage, even if the remainder of the body was in the rear portion, this was scored as being at the front). Affective behaviors were summed within and across bins to generate a continuous index of affective reactivity for each animal during each trial.

### Statistical analysis

R version 4.0.4^80^ was used for statistical analyses. Data were analyzed using linear mixed-effects (LME) models in *lme4*^81^ fit by maximum likelihood (Laplace Approximation); subject ID was included as a random effect in all models. Follow-up LME models were fit to determine group differences in particular test conditions. Planned comparisons were made using *emmeans*^82^. To limit the number of comparisons, two were set a priori: (1) individually-housed monkeys were compared to all of the socially-housed monkeys (i.e., grate-, intermittently-, and continuously-paired) combined and (2) grate-paired monkeys were compared to all of the full-contact monkeys (i.e., intermittently- and continuously-paired) combined. A multi-variate *t* adjustment was applied. For models that included only three social housing conditions (i.e., ORT models that evaluated responses to complex objects, which did not include grate-paired monkeys), only one comparison was made: individually-housed monkeys were compared to socially-housed monkeys (i.e., intermittently- or continuously-paired). Latency data were analyzed using a survival analysis (mixed effects Cox model implemented in *coxme*^83^) with a censored latency of 30 s. Difference scores were computed and analyzed for each animal using the HIT *stare near* and *stare far* data to investigate group differences across conditions. Importantly, analyses carried out via these procedures are relatively insensitive to unequal sample sizes^84^.

Analysis of monkeys’ ages at the time of testing revealed that there were significant differences in age across social housing conditions at the beginning of HIT, but not at the beginning ORT (see Supplementary Results; Supplementary Fig. 1A,B). As a result, we included age in the HIT models. Although previous research has not investigated age-related effects on ORT outcomes per se, one study showed that social housing conditions, and not age, impacted monkeys’ tendency to manipulate novel objects^85^. Given this, and the lack of significant group differences in age for ORT, we did not include age in our ORT models. There were also significant differences across samples in terms of how much time they had spent indoors prior to testing (see Supplementary Results; Supplementary Fig. 1C,D) and so this variable was included as a fixed effect in all models to be certain that social housing effects could not be explained by other means. Neither age nor duration of previous indoor housing were significant predictors in any of our analyses (all *p* > 0.05). As such, we do not report these effects throughout the “Results”.

## Results

### Experiment 1: object responsivity test

#### Food retrieval

**Figure 1:**
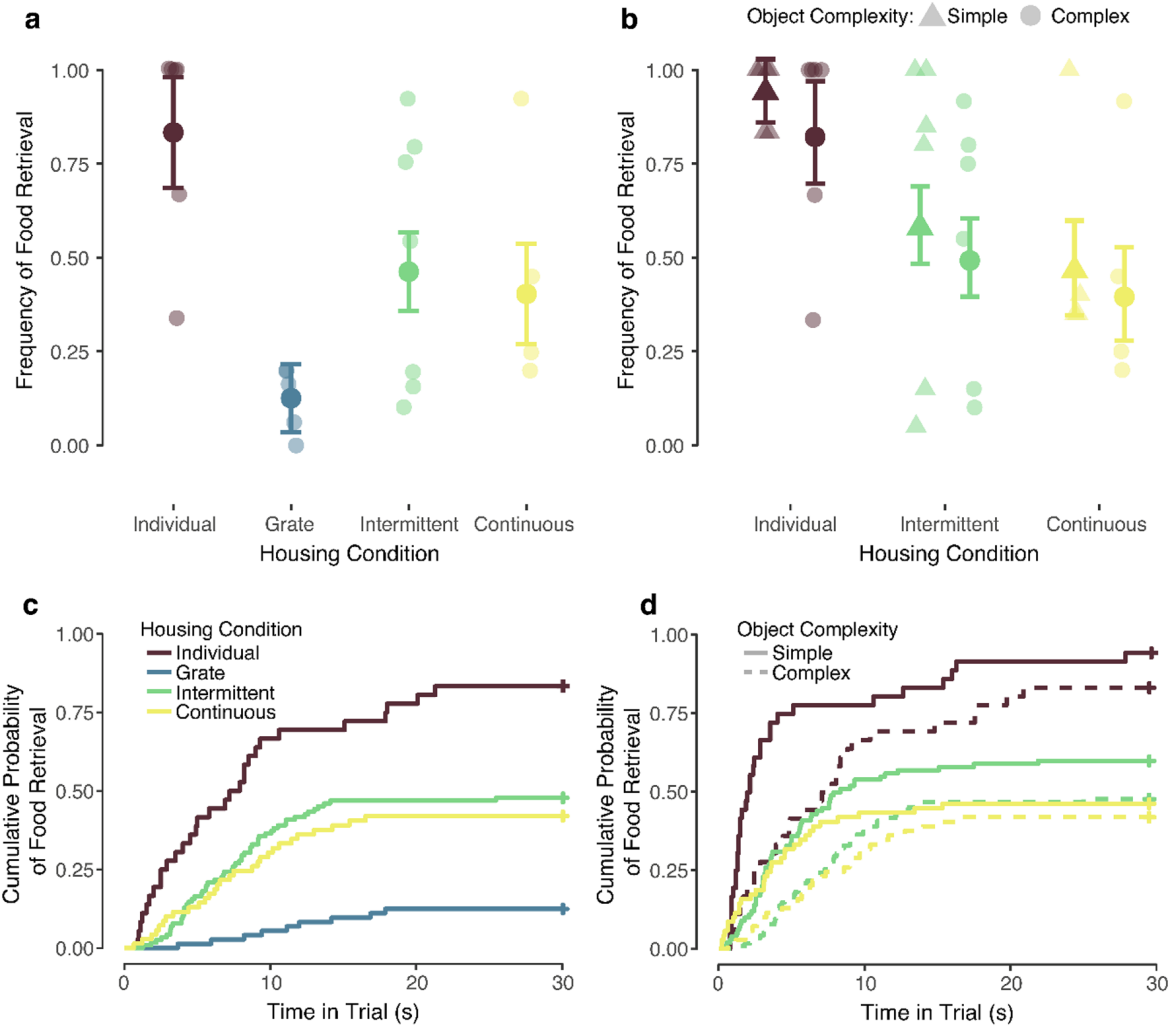
Food retrieval during the Object Responsivity Test. (**a**) Retrieval frequency during complex object trials for all monkeys. Individually-housed monkeys retrieved food significantly more frequently than socially-housed monkeys and grate-paired monkeys retrieved food significantly less frequently than those housed in full-contact. (**b**) Retrieval frequency during simple (triangle) and complex (circle) object trials for monkeys who completed both trial types. Groups did not differ significantly in retrieval frequency when both levels of object complexity were included. Means ± adjusted 95% confidence intervals and individual data are shown. (**c**) Cumulative probability of food reward retrieval as a function of time elapsed in trial during complex object trials. Individually-housed monkeys retrieved food significantly faster than socially-housed monkeys and grate-paired monkeys retrieved food significantly slower than monkeys housed in full-contact. (**d**) Cumulative probability of food reward retrieval as a function of time elapsed in trial during simple (solid line) and complex (dashed line) object trials for monkeys who completed both trial types. Individually-housed monkeys retrieved food significantly faster than socially-housed monkeys in the presence of complex, but not simple, objects. Survival curves are shown. Retrieval latencies of 30 s (no retrieval) are right-censored.

Retrieval frequencies during complex object trials were analyzed first as all subjects completed these trials. We fit a generalized linear model with housing condition and duration of previous indoor housing as fixed effects and trials nested within subjects as a random effect. There was a significant effect of housing condition on frequency of food retrieval (*χ*^2^(3) = 14.74, *p* = 0.002). Custom contrasts (see “Methods”) were used to compare the estimated marginal means across housing conditions and the multi-variate *t* adjustment was applied. Individually-housed monkeys retrieved food significantly more frequently than socially-housed monkeys (i.e., grate-, intermittently-, or continuously-paired; *p* = 0.009) and grate-paired monkeys retrieved food significantly less frequently than monkeys housed in full-contact (i.e., intermittently- or continuously-paired; *p* = 0.04) (see Fig. 1a).

Food retrieval frequencies during simple object (i.e., featureless objects like wooden blocks) and complex object (i.e., information-rich objects like animal figurines or household objects) trials were analyzed for monkeys who completed both trial types. We again fit a generalized linear model, this time with housing condition, object complexity (i.e., simple or complex), and duration of previous indoor housing as fixed effects, a housing condition × complexity interaction, and trials nested within subjects as a random effect. As only one grate-paired monkey experienced both trial types, this animal was excluded from all analyses including both simple and complex objects. There was a significant main effect of complexity (*χ*^2^(1) = 4.93, *p* = 0.03) on food retrieval; all monkeys retrieved food more frequently during simple vs. complex object trials. The effect of housing condition was not significant (*χ*^2^(2) = 3.48, *p* = 0.18) and the interaction between housing condition and complexity was not significant (*χ*^2^(2) = 1.31, *p* = 0.52). Although the effect of housing condition was not significant, visual inspection of the data suggested a possible impact of social housing, which we may have been underpowered to detect (see Fig. 1b). Although not significantly, individually-housed monkeys retrieved food more frequently during both simple (Mean ± SD = 0.94 ± 0.25) and complex (Mean ± SD = 0.83 ± 0.40) object trials than intermittently- (simple: Mean ± SD = 0.59 ± 0.53; complex: Mean ± SD = 0.50 ± 0.54) or continuously-paired (simple: Mean ± SD = 0.47 ± 0.54; complex: Mean ± SD = 0.40 ± 0.53) monkeys.

We fit a mixed-effects Cox proportional hazards model on latency to retrieve the food reward during complex object trials. Housing condition also significantly influenced food retrieval latency (*χ*^2^(3) = 16.39, *p* < 0.001) during these trials. Individually-housed monkeys retrieved food significantly faster than socially-housed monkeys (*p* = 0.007) and grate-paired monkeys retrieved food significantly slower than monkeys housed in full-contact (*p* = 0.02) (see Fig. 1c). We fit an additional mixed-effects proportional hazards model on latency to retrieve the food reward during both simple and complex object trials, including a housing condition × complexity interaction. When these trials were analyzed, there was a significant main effect of object complexity (*χ*^2^(1) = 38.40, *p* < 0.001). The effect of housing condition failed to reach the conventional level of significance (*χ*^2^(3) = 6.65, *p* = 0.08). There was, however, a significant interaction between housing condition and complexity (*χ*^2^(3) = 12.25, *p* = 0.007). Individually-housed monkeys retrieved food significantly faster than monkeys paired in full-contact in the presence of complex objects (*p* = 0.01), but not simple objects (*p* = 0.18). All groups retrieved the food faster during simple vs. complex object trials (see Fig. 1d).

#### Affective reactivity

**Figure 2:**
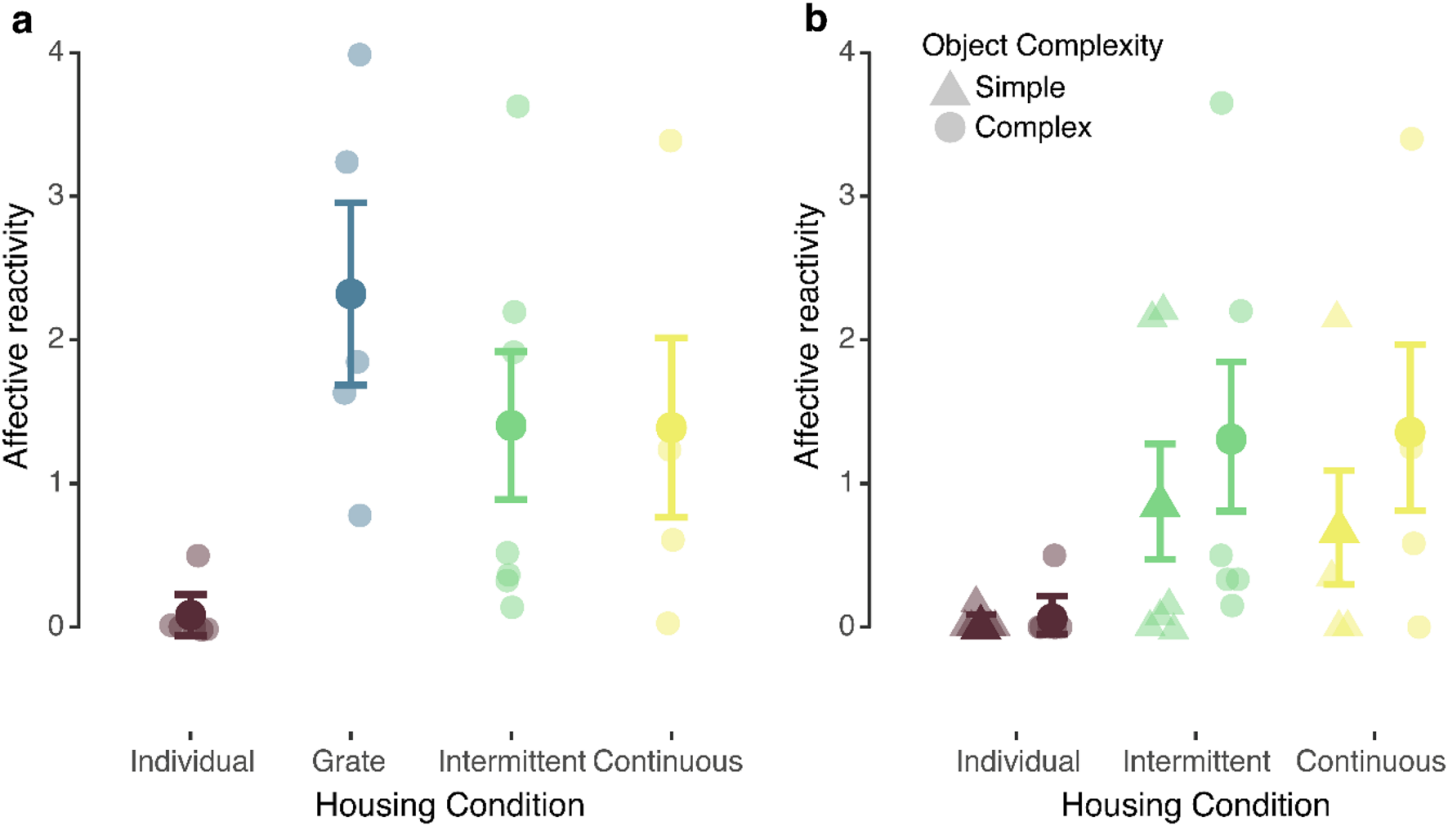
Affective reactivity during the Object Responsivity Test. (**a**) Affective reactivity (combined frequency of all affective behaviors) during complex object trials. Individually-housed monkeys were significantly less reactive than socially-housed monkeys. (**b**) Affective reactivity during simple (triangle) and complex (circle) object trials for monkeys who completed both trial types. All monkeys were significantly more reactive to complex vs. simple objects. Groups did not differ significantly when both levels of complexity were considered. Means ± adjusted 95% confidence intervals and individual data are shown.

As described in the Methods, affective reactivity was computed for each subject during each trial by summing the frequencies of individual affective behaviors generated. We then fit a generalized linear model with housing condition and duration of previous indoor housing as fixed effects and trials nested within subjects as a random effect to assess affective reactivity during complex object trials. There was a significant effect of housing condition on affective reactivity to complex objects (*χ*^2^(3) = 13.27, *p* = 0.004). Individually-housed monkeys were less reactive to complex objects than socially-housed monkeys (*p* = 0.005) and grate-paired monkeys did not differ significantly from monkeys housed in full-contact (*p* = 0.21) (see Fig. 2a). We fit an additional generalized linear model with housing condition, object complexity, and previous duration of indoor housing as fixed effects, a housing condition × complexity interaction, and trials nested within subjects as a random effect to assess responses to both simple and complex objects. The main effect of complexity (*χ*^2^(1) = 13.96, *p* < 0.001) was significant. The effect housing condition (*χ*^2^(2) = 4.98, *p* = 0.08) failed to reach the conventional level of significance and the interaction between housing condition and complexity was not significant (*χ*^2^(2) = 0.60, *p* = 0.74). Despite the effect of housing condition failing to reach the conventional level of significance, visual inspection of the data suggested a potential impact of housing condition that we were underpowered to detect (see Fig. 2b). Individually-housed monkeys were less reactive during both trial types (simple: Mean ± SD = 0.03 ± 0.18; complex: Mean ± SD = 0.08 ± 0.39) than intermittently- (simple: Mean ± SD = 0.88 ± 2.07; complex: Mean ± SD = 1.33 ± 2.67) or continuously-paired (simple: Mean ± SD = 0.69 ± 1.69; complex: Mean ± SD = 1.39 ± 2.46) monkeys. All groups showed greater reactivity to complex as compared to simple objects.

### Experiment 2: human intruder test

#### Position in cage

**Figure 3:**
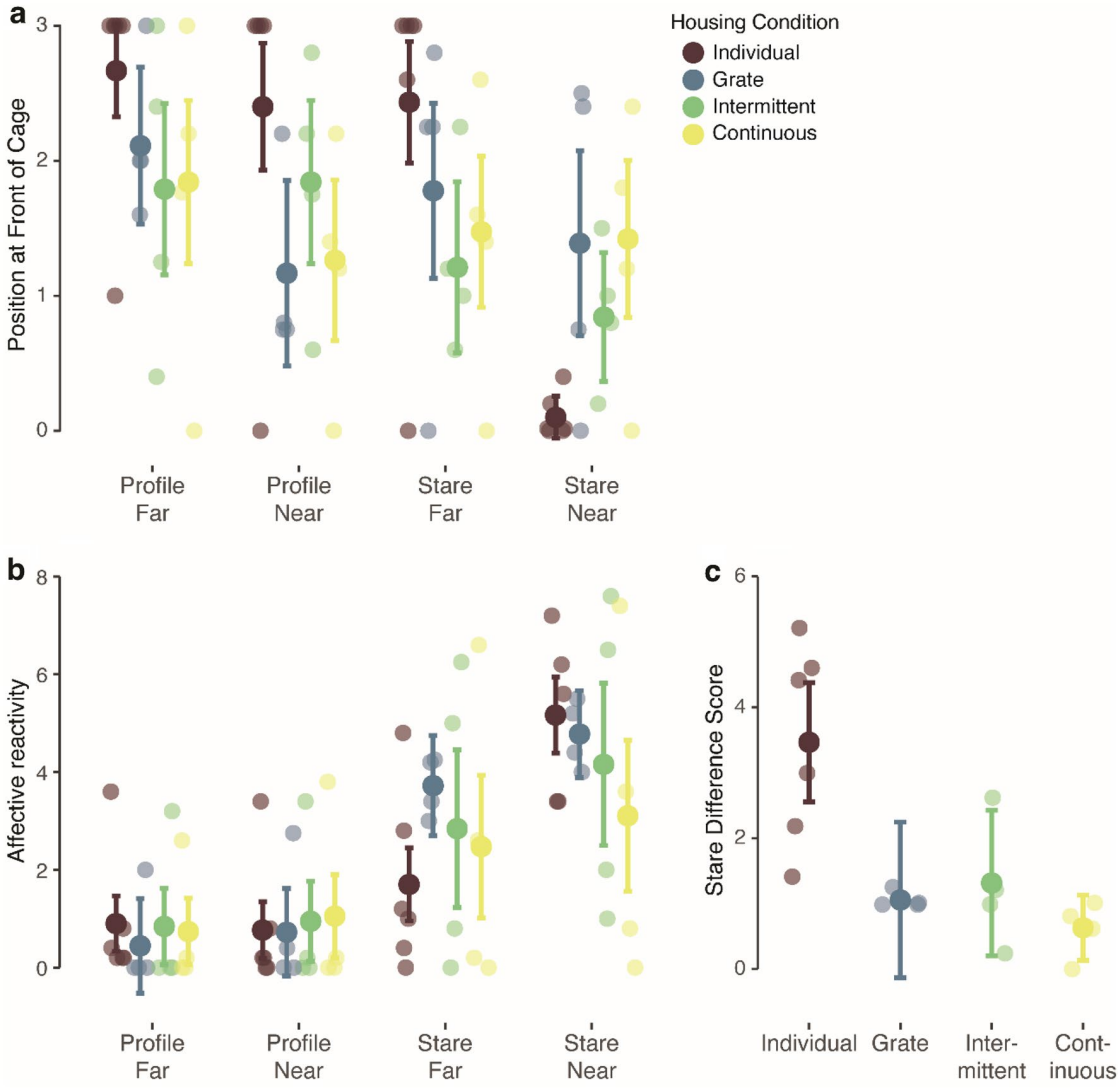
Responses during the Human Intruder Test. (**a**) Tendency to be present at the front of the cage (scored as the presence of the monkey’s head in the front or rear half of the cage) across condition for each group. Individually-housed monkeys spent less time at the front of the cage than socially-housed monkeys during stare near. (**b**) Affective reactivity (combined frequency of affective behaviors) across condition for each group. Groups did not differ significantly across conditions. (**c**) Affectivity reactivity difference scores (*stare near*–*stare far*) for each group. Individually-housed monkeys had significantly higher difference scores than socially-housed monkeys. Means ± adjusted 95% confidence intervals and individual data are shown.

We analyzed position in cage—determined by the presence of the monkey’s head either in the front or rear half of the cage—using a generalized linear model with housing condition, task condition (*profile far, profile near, stare far, stare near*), test day (days 1–5), age, and previous duration of indoor housing as fixed effects, housing condition × test day and housing condition × task condition interactions, and trials nested within subjects as a random effect. There was a significant main effect of task condition on position at front of cage (*χ*^2^(3) = 14.81, *p* = 0.002) such that monkeys spent more time at the front of the cage during the *profile* as compared to the *stare* conditions. The effects of test day (*χ*^2^(4) = 1.26, *p* = 0.87) and housing condition (*χ*^2^(3) = 1.23, *p* = 0.75) were not significant. There was a significant interaction of housing condition and task condition (*χ*^2^(9) = 33.97, *p* < 0.001). Comparison of the estimated marginal means revealed significant differences between the individually-housed and socially-housed monkeys only in the *stare near* condition (*p* < 0.001; *profile far*: *p* = 0.37; *profile near*: *p* = 0.54; *stare far*: *p* = 0.50). Individually-housed monkeys spent less time at the front of the cage during this condition than socially-housed monkeys (see Fig. 3a). Grate-paired monkeys did not differ significantly from monkeys housed in full-contact in any of the conditions (*profile far*: *p* = 0.70; *profile near*: *p* = 0.37; *stare far*: *p* = 0.81; *stare far*: *p* = 0.79).

#### Affective reactivity

We analyzed affective reactivity using an identical model to that specified for position in cage. There were significant main effects of test day (*χ*^2^(4) = 17.20, *p* = 0.002) and task condition (*χ*^2^(3) = 283.79, *p* < 0.001) on affective reactivity to the human intruder. The main effect of housing condition was not significant (*χ*^2^(3) = 0.22, *p* = 0.97). The interaction between test day and housing condition was not significant (*χ*^2^(12) = 19.79, *p* = 0.07). However, the interaction between housing condition and task condition was significant (*χ*^2^(9) = 29.27, *p* < 0.001) (see Fig. 3b). All groups showed declining reactivity across test days and increased reactivity in the *stare* conditions relative to the *profile* conditions.

To understand what was driving the significant interaction between housing condition and task condition, we analyzed responses in the two *profile* and two *stare* conditions separately, as monkeys are often lowly reactive during *profile*^78^. As expected, reactivity was low during *profile*, so there was not a significant effect of housing condition (*χ*^2^(3) = 2.55, *p* = 0.47), test day (*χ*^2^(4) = 2.29, *p* = 0.68) or distance (*χ*^2^(1) = 0.52, *p* = 0.47), nor was the interaction between housing condition and distance (*χ*^2^(3) = 1.71, *p* = 0.55) significant. There was a significant interaction between housing condition and test day (*χ*^2^(12) = 27.96, *p* = 0.006). Comparison of the estimated marginal means revealed that grate-paired monkeys were significantly less reactive on testing day 2 than monkeys housed in full-contact (*p* = 0.02). Grate-paired and full-contact groups did not differ significantly on any other days (all *p* > 0.05) and individually- and socially-housed monkeys did not differ significantly on any test day (all *p* > 0.05). When reactivity during *stare* trials was considered, there were significant main effects of test day (*χ*^2^(4) = 14.64, *p* = 0.006) and distance (*χ*^2^(1) = 37.19, *p* < 0.001), but not housing condition (*χ*^2^(3) = 0.14, *p* = 0.99). Critically, here, the interaction between housing condition and distance was significant (*χ*^2^(3) = 19.29, *p* < 0.001). Comparison of the estimated marginal means did not reveal a significant difference between individually- and socially-housed monkeys in either *stare far* (*p* = 0.87) or *stare near* (*p* = 0.84) and grate-paired monkeys also did not differ significantly from those housed in full-contact in either condition (*stare far*: *p* = 0.98; *stare near*: *p* = 0.97). Individually-housed monkeys appeared to be more reactive in *stare near* than *stare far*, while socially-housed monkeys showed similar reactivity across conditions. To investigate this, we computed difference scores between *stare near* and *stare far* responses for each monkey. Analysis of the difference scores revealed a significant effect of housing condition (*χ*^2^(3) = 12.81, *p* = 0.005). Individually-housed monkeys had significantly higher difference scores than socially-housed monkeys (*p* = 0.01). The difference scores of grate-paired monkeys did not differ significantly from monkeys housed in full-contact (*p* = 0.34) (see Fig. 3c).

## Discussion

Despite many decades of use in neuropsychological experiments, the social contexts in which adult monkeys live and the impacts that these contexts may have on behavioral responses in tasks commonly used to assess psychosocial behavior have historically gone unacknowledged. Our data demonstrate that social context matters for affective reactivity. Adult monkeys housed in different social conditions responded differently in the very same tasks that behavioral neuroscientists and psychologists use to model neuropsychiatric disorders^86–90^ and determine the neural correlates of affective processing^54,55,58,59,63,75,91^. Adult monkeys housed individually during experiments generated different affective responses to threat compared to monkeys with social contact (either grate-, intermittently- or continuously-paired). Patterns of behaviors were consistent across experiments relative to housing condition.

When presented with ostensibly threating objects, individually-housed monkeys’ affective responses were dampened; they retrieved food rewards from beside objects faster, more frequently, and while generating fewer affective behaviors than socially-housed monkeys. Similarly, during HIT, isolated monkeys were less reactive and more willing to spend time at the front of the cage than socially-housed monkeys in three conditions. In the 4th condition, *stare near,* socially isolated monkeys exhibited the greatest affective reactivity, suggesting a different threshold for response than socially-housed monkeys. Importantly, there was variance in responses across ostensibly social contexts as well. Monkeys with restricted social access (grate-pairing) behaved differently from monkeys with full physical contact; they retrieved food rewards slower, less frequently, and while generating fewer behaviors during ORT. During HIT, however, grate-paired monkeys behaved comparably to those housed in full-contact, suggesting a more subtle impact on affective responding than isolation.

Our data demonstrate that social housing conditions during adulthood shape affective responding in a way that may be consistent with some of the impacts of variation in socialization in early development. One of the most indelible findings in comparative psychology is the demonstration that social isolation in infancy causes lasting disruptions to normal patterns of social and exploratory behaviors, originally observed by Harlow and colleagues^31,92,93^. While many studies of social restriction in infancy focus on the impact of rearing baby monkeys in nurseries without their mothers (typically with humans, or with other caretakers, such as dogs in^94^), accumulating evidence demonstrates that infants raised alone with their mothers or with another mother-infant pair also have disrupted behavior and physiology (e.g., increased heart rates and lower respiratory sinus arrhythmia^95^; immune response reductions^96^). Just as we found blunted reactivity to threat in individually-housed adult monkeys, Gottlieb and Capitanio^79^ found that nursery-reared infants had significantly lower “aggression” (including threats and vocalizations) and “displacement” (including yawns and tooth grinding) scores than outdoor socially reared-infants when responses to a threatening human intruder were assessed. Consistent with this and our results, Shannon et al.^97^ also found that mother-reared infants exhibited higher cortisol levels than nursery-reared infants (i.e., those raised with inanimate surrogate mothers) in response to examination and handling by a human experimenter, suggesting that socially isolated rearing blunts reactivity. Additional research is needed in which identical methodologies are used to make behavioral assessments of monkeys socially isolated during rearing or during adulthood, but our data suggest that social contexts in adulthood may have similar impacts on psychosocial outcomes that have simply gone uninvestigated because of norms related to husbandry in behavioral neuroscience.

One interpretation of the behavioral differences that we see in individually-housed monkeys is that they may be exhibiting a depression-like phenotype, which is potentially consistent with the human literature indicating that loneliness is a significant variable impacting depression (e.g., see^98^ for a meta-analysis). The emotion context-insensitivity (ECI) hypothesis of depression^99^ posits that depressed individuals exhibit diminished emotional reactivity to negative stimuli^100^, as we show in individually-housed monkeys. Additional research is needed to determine whether individually-housed monkeys also exhibit diminished affective reactivity to positive stimuli, which represents the other major component of the ECI hypothesis. Human studies also indicate a relationship between depression and impulsivity^101,102^, with depressed individuals showing increased impulsivity across three dimensions: behavioral loss of control, non-planning, and cognitive impulsivity^103^. Such a relationship may explain why individually-housed monkeys in our sample retrieved food rewards from beside threatening objects quicker and more frequently than socially-housed monkeys. A limitation of our study is that we do not also have home cage behavioral observations from these monkeys. As such, we could not determine whether individually-housed monkeys exhibited depression-like behaviors in this setting, including hunched posture, laying down, and increased day time sleep—behaviors that have previously been shown to increase when monkeys were relocated from social to non-social housing^43,44^.

Our data also make clear that not all social contexts are created equal. Restricted social access (grate-pairing) appears to be different from social housing conditions permitting at least some full-contact with a partner. During complex object trials, grate-paired monkeys were most reactive and retrieved the food reward less frequently than other socially-housed monkeys. This was the opposite pattern of results generated by isolated monkeys. One hypothesis is that grate-pairing causes an anxiety-like phenotype while isolation causes a depression-like phenotype, causing animals in those conditions to behave differently from the mean (or “normal”) in different ways. Prior studies assessing the differences between monkeys housed in protected contact (i.e., with a barrier allowing limited tactile contact, similar to our grate-paired monkeys) and those housed in full-contact have demonstrated significantly higher abnormal and anxiety-related behaviors during home cage observations for protected contact monkeys^104,105^. An additional study showed that monkeys housed in protected contact (in this case, much less restrictive contact, permitting monkeys the ability to reach entire limbs through to the other side of the cage) also exhibited significantly more motor stereotypic behaviors than monkeys housed in full contact^51^. While future research is needed to determine the mechanism behind this behavioral phenotype, this may be due in part to increased stress associated with the ability of pairs to display agonistic behaviors (e.g., threatening, cage-shaking; which do not require contact) in close proximity while affiliative behaviors (e.g., grooming; which does require close contact) were more challenging.

Many neuropsychiatric and behavioral neuroscience studies have used individually-housed monkeys, including studies probing the neural basis of socioaffective processing^60,62,64,70,106^ and of addiction or drug-seeking behaviors^107–111^. Even studies intending to specifically address depression induced through means other than social isolation (e.g., administration of immune cytokines^112^; social stress^113^) and those intending to determine the impact of early social deprivation^114^ or stress^115^, have all used at least some socially isolated subjects. In light of the findings we present here, the documented effects of these manipulations may be severely confounded by the impact of restricted social contact. Considering the potential impact of social context in such studies may provide a beneficial opportunity to resolve conflicts in the literature (e.g., the role of the amygdala in social cognition, as discussed in ^116^). Further, the impact of adult social conditions may extend beyond the study of psychosocial processes to other domains of psychology, including cognition, for which monkeys have been proven an essential model for the advancement of biomedical research^9,117^.

Our analyses were made possible by access to data from experiments that were carried out using nearly identical protocols in the same laboratory. Given methodological consistencies across studies, it is unlikely that the effects are due solely to differences in experimental techniques. Importantly, our subjects were all raised in conditions most closely resembling the natural rearing conditions of macaques as are possible in a captive setting through adulthood (i.e., ~ 4 years of age^118^). Subjects were chosen for these experiments because they exhibited species-typical behaviors, without abnormalities, when observed in the large outdoor cages that they were raised in prior to being moved indoors. It is therefore striking that we observed as strong of an impact of adult social condition as we did. Nevertheless, as slight procedural and other changes may have occurred over the course of time and the experiments we describe here, future studies should replicate the present experiments in monkeys housed in different social contexts (both between and within subjects) and tested concurrently by the same experimenters. Future studies should also address the potential role of sex in the impact of social housing conditions. The present study is limited in that we only report data from male monkeys. While male monkeys have been used preferentially in the historical literature, as more work is done in female monkeys the potential for such differences will be important to understand.

Much of what we know about the neural basis of psychological functions come from causal manipulations in nonhuman animals’ brains. If control animals in these studies are behaviorally abnormal, then these subjects do not provide a faithful baseline for comparison of the experimental conditions. Further, the conditions that drive this altered phenotype may interact with experimental manipulations, leading to incorrect inferences about the manipulation’s impact. While we demonstrate the impact of social context, other aspects including diet, husbandry practices, and features of experimenters may drive similar differences. We aim to initiate a conversation regarding the potential impact of social context on the results of studies aiming to reveal the nature or pathophysiology of socioaffective processing, including the potential for rethinking existing inferences in the literature. We call on other researchers to increase the transparency and specificity with which they describe the social contexts their subjects are housed in, urge them to house animals in full-contact whenever possible, and compel individuals reviewing the published literature to carefully consider social context when assessing the strength or validity of prior studies and corresponding inferences.

## Supplementary Information

Supplementary Information.

## Data Availability

All data are available at: https://osf.io/n5ym2/.
