# Supplementary material for: Social housing status impacts rhesus monkeys’ affective responding in classic threat processing tasks
Source: Sci Rep. 2022 Mar 9;12:4140. doi: 10.1038/s41598-022-08077-4 (PMC8907189; doi:10.1038/s41598-022-08077-4)
Supplement: Supplementary file 1 — Supplementary Information. [file 41598_2022_8077_MOESM1_ESM.docx]

**Supplementary Results**

*Age at Testing*

*Object Responsivity Test.* We assessed whether there were significant differences in subject ages at the time of ORT such that we could appropriately account for this variable in our analyses if necessary. A one-way ANOVA determined there was not a significant difference between ages at the start of ORT across social housing conditions (*F*(3,18)=2.38, *p*=0.10) (see Supplementary Fig. 1A). We also assessed whether there were significant differences in the amount of time subjects had previously lived indoors at the start of ORT. A one-way ANOVA determined that there was a significant difference between previous durations of indoor housing across social conditions (*F*(3,18)=4.72, *p*=0.01). Pairwise comparisons of the estimated marginal means revealed that individually-housed monkeys had been indoors for significantly less time than continuously-paired monkeys (*p*=0.009). All other comparisons were not significant (*p*>0.05) (see Supplementary Fig. 1B).

*Human Intruder Test.* We similarly assessed whether there were group differences in age at the start of HIT. A one-way ANOVA revealed a significant difference in age across social housing conditions (*F*(3,14)=14.47, *p*<0.001). Pairwise comparisons revealed that the individually-housed monkeys were significantly older than grate- (*p*=0.005), intermittently- (*p*=0.0007), or continuously-paired (*p*=0.0003) monkeys. All other comparisons were not significant (*p*>0.05) (see Supplementary Figure 1C). When we assessed group differences in duration of previous indoor housing at the start of HIT, a one-way ANOVA revealed that there were significant group differences (*F*(3,14)=26.77, *p*<0.001). Pairwise comparisons showed that individually-housed monkeys had spent significantly more time indoors as compared to intermittently- (*p*=0.01) or continuously-paired (*p*<0.001) monkeys. Continuously-paired monkeys had also spent significantly less time indoors than intermittently- (*p*=0.01) or grate-paired (*p*<0.001) monkeys. Intermittently-paired monkeys had spent significantly less time indoors than grate-paired monkeys (*p*=0.007). Individually-housed and grate-paired monkeys did not differ significantly (*p*=0.95) (see Supplementary Fig. 1D).


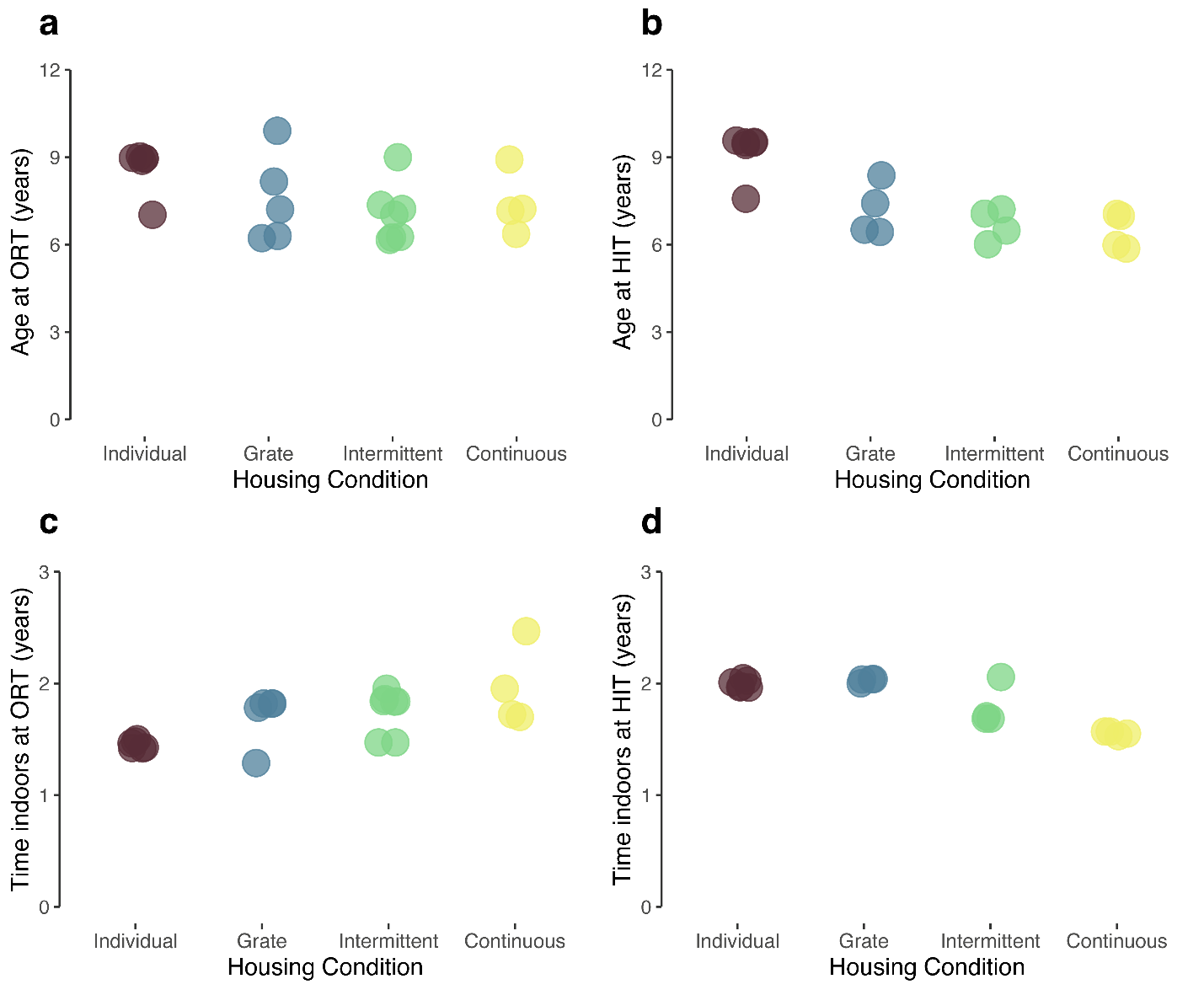


**Supplementary Figure 1.**

Ages and previous durations of time spent indoors at the time of experiments. (**A**) Subject ages in years at the start of the Object Responsivity Test. (**B**) Subject ages in years at the start of the Human Intruder Test. (**C**) Duration of time previously spent indoors in years at the start of the Object Responsivity Test. (**D**) Duration of time previously spent indoors in years at the start of the Human Intruder Test.

| Table S1 |  |
| --- | --- |
| *Behavioral Ethogram for the Object Responsivity Test* | |
| Behavior | Description |
| Affective behaviors |  |
| Lipsmack | Rapid lip movement usually with pursed lips and accompanied by a smacking sound |
| Grimace | Exaggerated grin with teeth showing |

**Table S1.**

Behavioral ethogram describing the two affective behaviors scored across all iterations of the *Object Responsivity Test*. Frequencies of these behaviors were summed to represent affective reactivity. Behavioral ethograms varied across studies but these two behaviors were recorded in all studies and thus used as our behavioral index. We note that while lipsmack is often interpreted as reflecting a positive affective state and grimace as reflecting a negative affective state, these behaviors both occur in a variety of contexts (social and non-social) with different affective valence.

| Table S2 |  |
| --- | --- |
| *Behavioral Ethogram for the Human Intruder Test* | |
| Behavior | Description |
| Affective behaviors |  |
| Lipsmack | Rapid lip movement usually with pursed lips and accompanied by a smacking sound |
| Grimace | Exaggerated grin with teeth showing |
| Threat | At least two of the following: open mouth stare, head-bob, ear slaps, bark vocalizations, or lunges |
| Cage Shake | Grasping of cage parts and shaking |
| Tooth Grind | Repetitive, audible rubbing of upper and lower teeth |
| Yawn | Open mouth, exposing teeth |
| Position in cage |  |
| Front vs. Back | Scored by location of the animal’s head in the front half or the back half of the cage |

**Table S2**

Behavioral ethogram describing the six affective behaviors scored across all iterations of the *Human Intruder Test*. Frequencies of these behaviors were summed to represent affective reactivity. Position in cage was considered separately. Behavioral ethograms varied across studies but these behaviors were recorded in all studies and thus used as our behavioral indices.
